# ^18^F-fluorodeoxyglucose positron emission tomography–computed tomography for assessing organ distribution of stressed red blood cells in mice

**DOI:** 10.1038/s41598-021-82100-y

**Published:** 2021-01-28

**Authors:** Wen-yu Yin, Jiao Yuan, Zhi-min Zhang, Cheng Mei, Wei Xu, Yong-xiang Tang, Fang Peng, Ning Li

**Affiliations:** 1 Department of Blood Transfusion, Clinical Transfusion Research Center, Xiangya Hospital, Central South University, Changsha, 410008 Hunan Province China; 2 Department of Infectious Diseases, Xiangya Hospital, Central South University, Changsha, 410008 Hunan Province China; 3 Clinical Laboratory, Xiangya Hospital, Central South University, Changsha, 410008 Hunan Province China; 4 Department of PET Center, Xiangya Hospital, Central South University, Changsha, 410008 Hunan Province China; 5 NHC Key Laboratory of Cancer Proteomics, Xiangya Hospital, Central South University, Changsha, 410008 Hunan Province China

**Keywords:** Immunology, Physiology

## Abstract

Red blood cells (RBCs) stressed by high temperature are similar to senescent or damaged RBCs in pathological conditions. RBCs can be efficiently labelled with ^18^F-fluorodeoxyglucose (FDG). The aim of this study was to assess stressed RBCs erythrophagocytosis and organ distribution in vivo with the application of ^18^F-FDG PET/CT. RBCs were induced under high temperature (48 °C) to prepare stressed RBCs. Fluorescence-activated cell sorting (FACS) was used to analyse reactive oxygen species (ROS) generation, intracellular Ca^2+^ concentration and membrane phosphatidylserine (PS) externalization of RBCs. ^18^F-FDG was used to label RBCs and assess the erythrophagocytosis. Finally, ^18^F-FDG PET/CT was applied to reveal and measure the organ distribution of stressed RBCs in mice. Compared with untreated RBCs, stressed RBCs decreased in cell volume and increased in ROS level, intracellular Ca^2+^ concentration, and PS exposure. RBCs could be labelled by ^18^F-FDG. Stressed RBCs tended to be phagocytosed by macrophages via assessment of FACS and radioactivity. ^18^F-FDG PET/CT imaging showed that stressed RBCs were mainly trapped in spleen, while untreated RBCs remained in circulation system. Thus, stressed RBCs can be effectively labelled by ^18^F-FDG and tend to be trapped in spleen of mice as assessed by PET/CT.

## Introduction

RBCs are the main component of blood, accounting for half of the total blood volume. Under physiological conditions, RBCs have biconcave shape and exhibit reversible deformability and durability^1^. Since RBCs have a simple structure, lacking nuclei and internal organelles, they are highly susceptible to various changes in the internal environment, such as oxidative stress, osmotic shock, energy depletion, and cytokines. These changes lead to morphological and functional alterations of RBCs, including lipid oxidation imbalance, aberrant activation of membrane ion channels, phosphatidylserine (PS) externalization, and aggregation or allostery of membrane proteins^2–4^, eventually accelerating the clearance of RBCs. Several studies show that these changes occur in RBCs in some chronic diseases^5–7^. Macrophages remove senescent or damaged RBCs from the circulation by a fundamental physiological process called erythrophagocytosis, which is essential for iron/heme metabolism and homeostasis. It is generally believed that erythrophagocytosis is executed by hepatic and splenic macrophages^8,9^. However, which kind of macrophages have a key function in RBCs clearance remains elusive. Some studies demonstrated that storage-damaged and senescent RBCs are mainly cleared by splenic red pulp macrophages^10,11^, while other researchers consider hepatic macrophages as the vital cells in the clearance of aged and stressed RBCs^12,13^. Therefore, it is of great value to study the distribution of RBCs in pathological state and the main scavenging organs.

As one of the most advanced technologies, positron emission tomography–computed tomography (PET/CT) can obtain metabolic and anatomical data simultaneously. PET/CT uses an injection of short half-time radioactive isotope labelled substance into the body to reflect the status of metabolism activities through the aggregation of radioactivity. ^18^F-fluorodeoxyglucose (^18^F-FDG), a radioactively labelled glucose analogue, is the main clinical PET/CT imaging agent. ^18^F-FDG PET/CT has been widely applied in the detection and diagnosis of diseases with high glucose metabolism including infection/inflammation and cancer, by imaging relative ^18^F-FDG uptake rates in various tissues^14^. The energy metabolism of RBCs relies on glycolysis. As a glucose analogue, ^18^F-FDG can thus be taken up by RBCs to achieve RBC labelling, which was recently shown to be feasible^15^. Currently, whether stressed RBCs are phagocytosed and where they are distributed in vivo remains unclear.

In this study, we used high temperature-induced stressed RBCs to simulate RBCs in pathologic conditions. ^18^F-FDG was used to label RBCs to detect erythrophagocytosis and PET/CT was performed to assess organ distribution of stressed RBCs in mice.

## Results

### Characteristics of stressed RBCs

**Figure 1:**
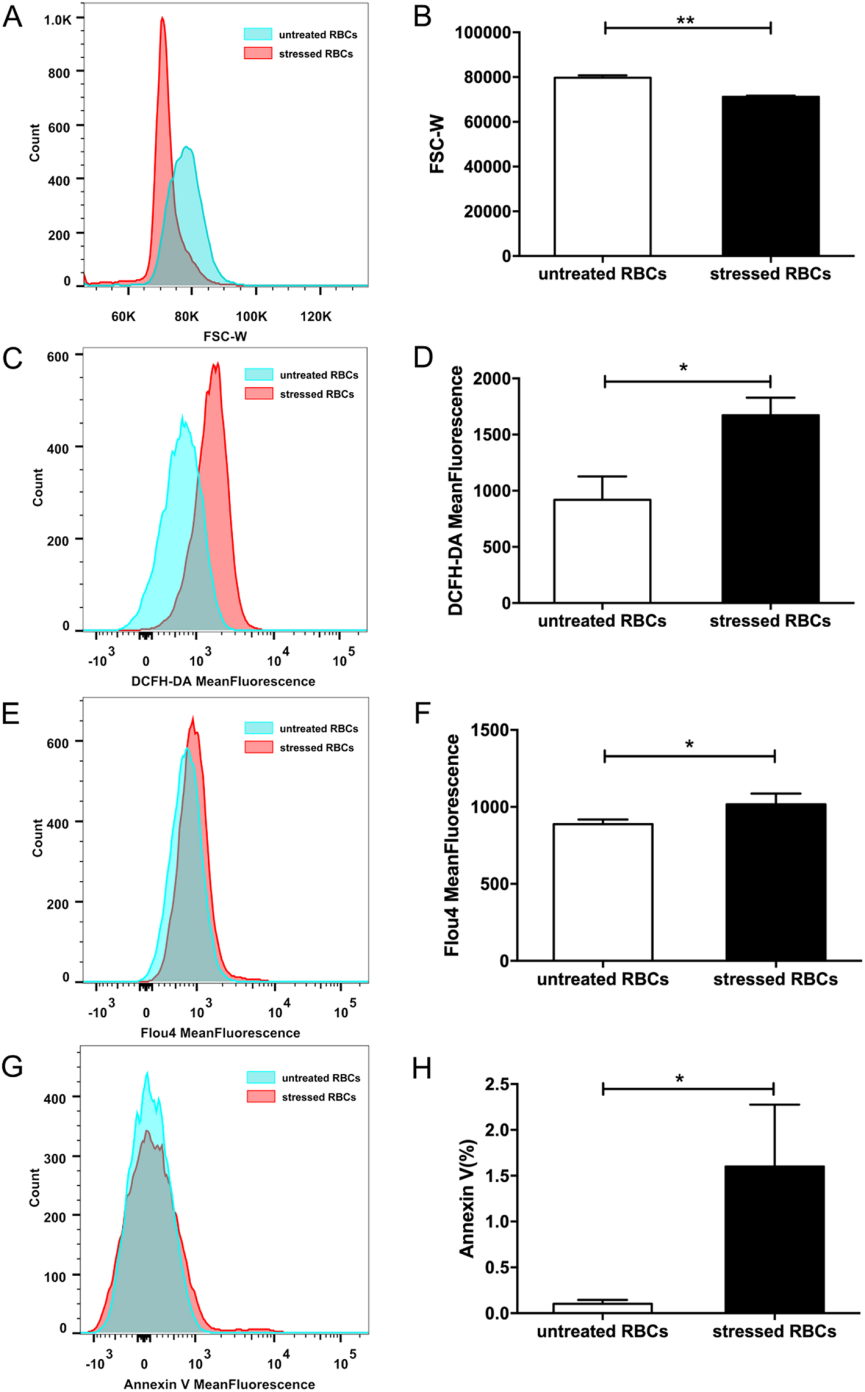
Characteristics of stressed RBCs. RBCs obtained from 8-week-old BALB/c mice were incubated at 48 °C for 30 min or kept at room temperature for 30 min (n = 9). (**A**,**B**) Cell volume of stressed RBCs and untreated RBCs. Cell volume was represented by forward scatter width. (**C**,**D**) ROS level of stressed RBCs and untreated RBCs. (**E**,**F**) Intracellular Ca^2+^ concentration of stressed RBCs and untreated RBCs. (**G**,**H**) PS externalization of stressed RBCs and untreated RBCs (*p < 0.05; **p < 0.01).

After heating for 30 min at 48 °C, RBCs changed in morphology. Forward scatter can reflect the relative volume of RBCs^16^, the forward scatter width (FSC-W) of untreated RBCs was higher than FSC-W of stressed RBCs, indicated that cell volume was smaller in stressed RBCs than in untreated RBCs (Fig. 1A,B). The membrane molecular distribution and intracellular ion concentration of stressed RBCs also differed from untreated RBCs. ROS level and intracellular Ca^2+^ concentration was described as mean fluorescence intensity (MFI), PS externalization was expressed as Annexin-positive percentage. The DCFH-DA MFI (Fig. 1C,D), Flou4 MFI (Fig. 1E,F) and Annexin-positive percentage (Fig. 1G,H) of stressed RBCs were higher than that of untreated RBCs. These results indicated that heating resulted in elevated ROS levels, increased calcium influx, and higher PS exposure in stressed RBCs.

### Stressed RBCs tended to be phagocytosed

**Figure 2:**
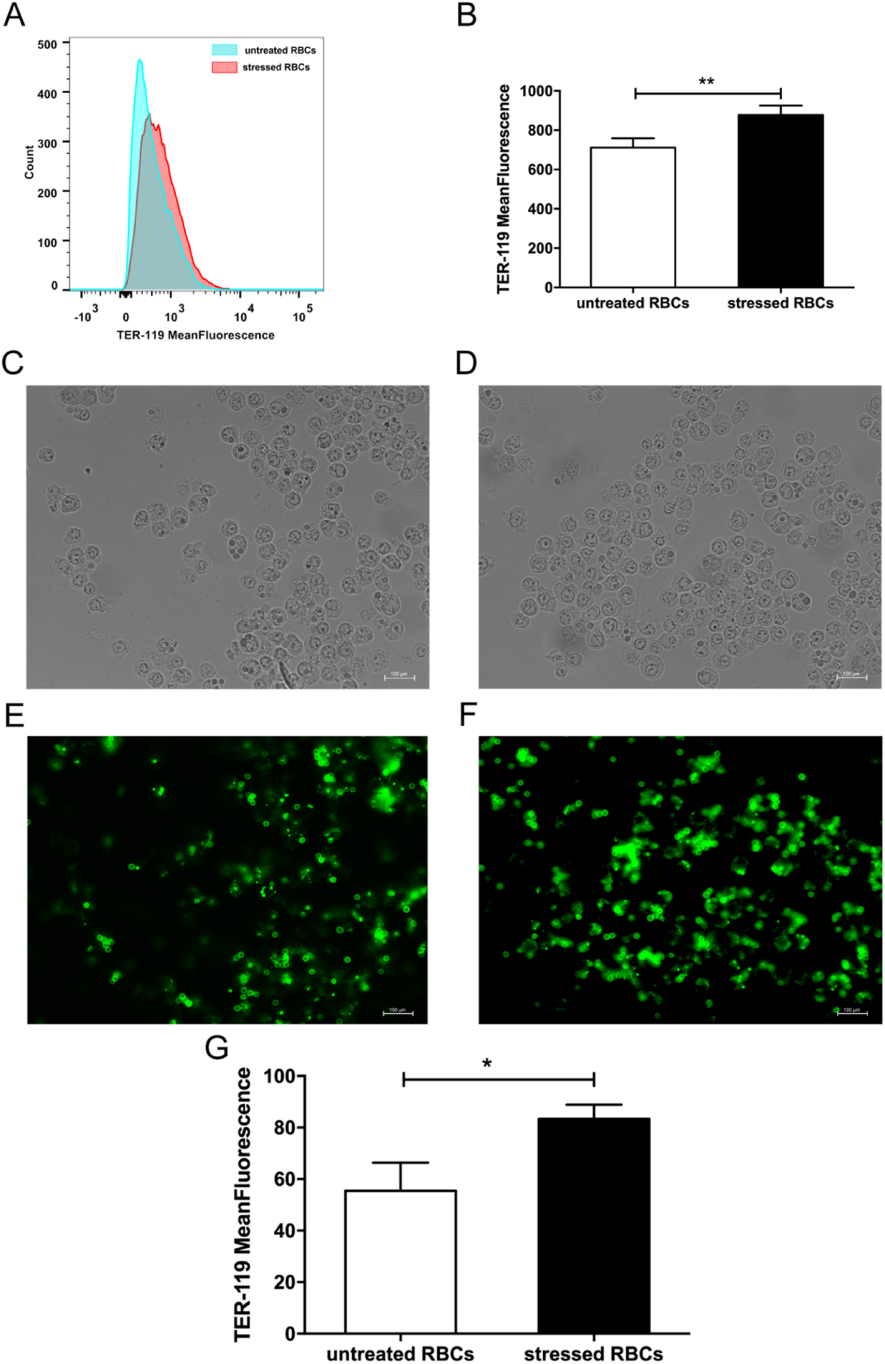
Stressed RBCs tended to be phagocytosed. FITC-TER 119-labelled RBCs were incubated with BALB/c macrophage cell line Raw 264.7 (n = 9). (**A**,**B**) FACS results of erythrophagocytosis. Erythrophagocytosis was represented by FITC-mean fluorescence intensity. (**C**,**F**) Immunofluorescence results of erythrophagocytosis. Bright field showed macrophages treated with untreated RBCs (**C**) and stressed RBCs (**D**), fluorescence field showed TER-119 labelled untreated RBCs (**E**) and stressed RBCs (**F**). Arrow showed RBCs located in macrophages. (**G**) statistic results of erythrophagocytosis of untreated RBCs and stressed RBCs (*p < 0.05; **p < 0.01).

Since FACS is the most common method to measure erythrophagocytosis, we used fluorescence probe TER-119-labelled RBCs to detect this process. The FITC-dependent fluorescence intensity of macrophages reflected the phagocytosis of RBCs. The mean fluorescence intensity of macrophages incubated with stressed RBCs was higher than that of macrophages incubated with untreated RBCs (Fig. 2A,B). Consistent with the FACS results, immunofluorescence indicated that stressed RBCs were more likely to be engulfed by macrophages than untreated RBCs. Bright field showed Raw 264.7 cells (Fig. 2C,D). Fluorescence field showed that many stressed RBCs were located in macrophages, indicating they were phagocytosed (Fig. 2F). Untreated RBCs were less phagocytosed by macrophages, and less untreated RBCs adhered to macrophage membranes (Fig. 2E). The mean fluorescence intensity value of macrophages treated with stressed RBCs was higher than that of untreated RBCs (Fig. 2G).

**Figure 3:**
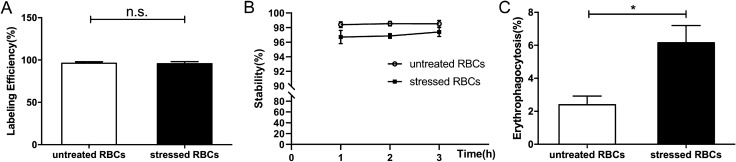
Erythrophagocytosis measured by ^18^F-FDG. (**A**) Labelling efficiency of untreated RBCs and stressed RBCs (n = 5). (**B**) Post-labelling stability of untreated RBCs and stressed RBCs (n = 5). (**C**) Phagocytosis ratio of untreated RBCs and stressed RBCs (n = 9) (*p < 0.05).

^18^F-FDG labelling efficiency of untreated and stressed RBCs was 96.13 ± 1.81% and 95.71 ± 2.46% (Fig. 3A). Difference between the labelling efficiency of stressed RBCs and untreated RBCs was not significant. The post-labelling stability of untreated RBCs were 98.91 ± 0.079%, 98.2 ± 0.183%, 98.34 ± 0.079% at 1 h, 2 h and 3 h respectively, and the labelling stability of stressed RBCs were 97.68 ± 0.452%, 96.56 ± 0.375%, 96.71 ± 0.478% (Fig. 3B). The radioactivity of the unphagocytic RBCs and macrophages were measured to calculate the ratio of phagocytosis. The ratio of stressed RBCs incubated macrophages was higher than the ratio of untreated RBCs incubated macrophages (Fig. 3C), which meant stressed RBCs tended to be phagocytosed by macrophages compared with untreated RBCs.

### Stressed RBCs were trapped in spleen

**Figure 4:**
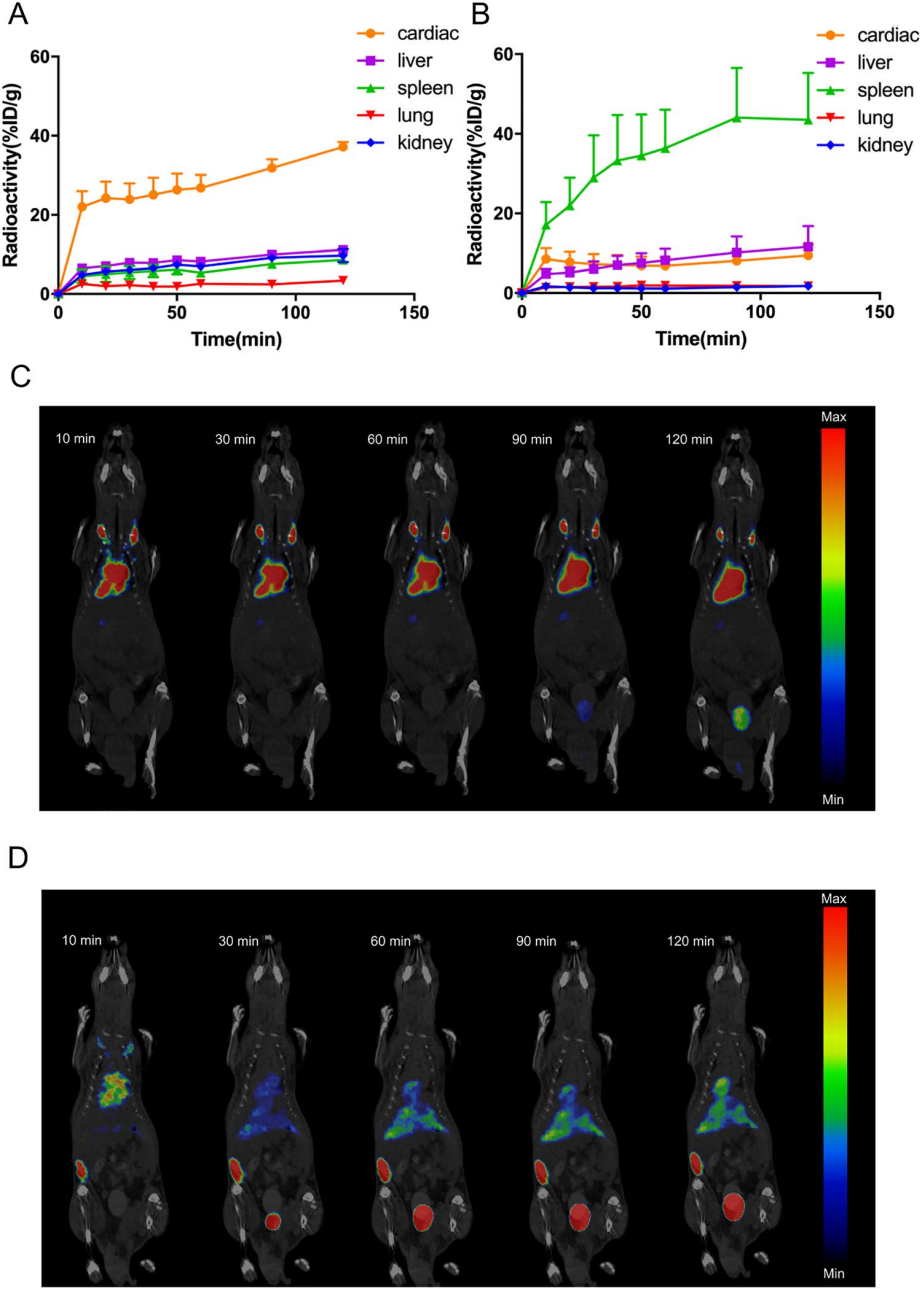
Stressed RBCs were trapped in spleen. (**A**,**B**) Time-activity curves of cardiac, liver, spleen, lung, and kidney of mice injected with ^18^F-FDG-labelled untreated RBCs (**A**) and stressed RBCs (**B**) during PET/CT imaging for 120 min (n = 3); (**C**,**D**) Representative serial images after intravenous injection for 120 min of ^18^F-FDG-labelled untreated RBCs (**C**) and stressed RBCs (**D**).

Upon injection with untreated RBCs, the cardiac radioactivity was high while the radioactivity of other organs were low (Fig. 4A). Images showed that untreated RBCs mainly distributed in cardiac (Fig. 4C). Instead, upon injection with stressed RBCs, the splenic radioactivity was high (Fig. 4B) and images revealed that spleen showed a strong accumulation of ^18^F-FDG-labelled stressed RBCs (Fig. 4D). In short, injected stressed RBCs were trapped in spleen while untreated RBCs kept in cardiovascular system.

**Figure 5:**
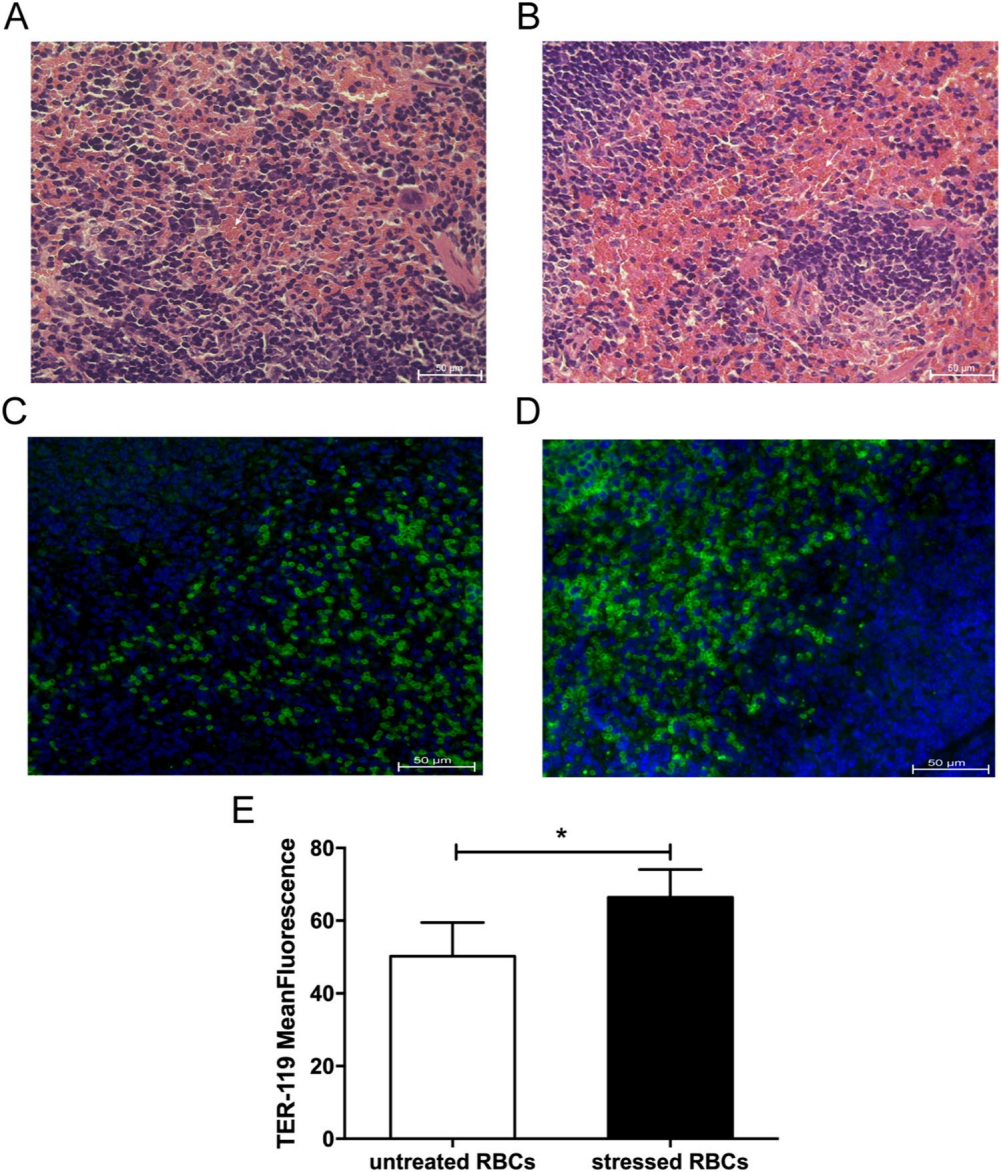
H&E stain and immunofluorescence of spleen sections (n = 4). (**A**,**B**) H&E stain of spleen of mice injected with untreated RBCs (**A**) and stressed RBCs (**B**). The white arrow shows RBCs in spleen. (**C**,**D**). immunofluorescence of spleen of mice injected with untreated RBCs (**C**) and stressed RBCs (**D**), green means RBCs. (**E**) The statistic results of immunofluorescence of spleen. (*p < 0.05).

Subsequently, H&E staining of spleen from stressed RBCs-injected mice showed many RBCs in splenic sinuses (Fig. 5B), but less RBCs in spleen of untreated RBCs-injected mice (Fig. 5A). Immunofluorescence also revealed that stressed RBCs gathered in spleen (Fig. 5C–E). These results demonstrated that stressed RBCs trapped in spleen, which were consistent with the data of in vivo organ distribution measured by PET/CT.

## Discussion and conclusion

There is controversy about the main organ responsible for RBC clearance^10–13^. Our study used PET/CT to confirm the accurate organ distribution of stressed RBCs, which could reflect their clearance site. First, we demonstrated that stressed RBCs showed similar characteristics to RBCs under pathological state (Fig. 1). Second, we proved that ^18^F-FDG can label RBCs effectively and there were no statistical differences between the labelling efficiency of stressed and untreated RBCs (Fig. 3A). Thus, ^18^F-FDG labelling of RBCs is suitable to assess erythrophagocytosis and in vivo organ distribution of stressed RBCs and untreated RBCs. Then, our data demonstrated increased erythrophagocytosis in stressed RBCs (Figs. 2, 3C). Finally, PET/CT imaging showed that splenic radioactivity increased rapidly in mice injected with stressed RBCs (Fig. 4D), and these results were confirmed by H&E staining and immunofluorescence of spleen sections (Fig. 5).

Spleen is a part of reticuloendothelial system which related to RBCs clearance. Stressed RBCs are trapped in spleen means stressed RBCs are likely removed by spleen. This could explain the anaemia in some diseases. Besides, injected stressed RBCs trapped in spleen indicate low efficiency of transfusion since RBCs must be in cardiovascular system to function properly. RBCs in cardiovascular system indicate that RBCs could perform their physiological functions, which means that transfusion could achieve its purpose. Thus, ^18^F-FDG PET/CT can contribute to investigate the transfusion efficiency.

In our study, we use ^18^F-FDG to label RBCs. As a glucose analogue, ^18^F-FDG could be taken up by RBCs. After ^18^F-FDG converted into ^18^F-FDG-P, it can’t continue the following glycolysis process until ^18^F undergoes radioactive decay^17^. The post-labelling stability of ^18^F-FDG labelled RBCs was high, which means ^18^F-FDG labelled RBCs was suitable for displaying RBCs distribution.

Our research used 48℃ treated RBCs (stressed RBCs) to simulate RBCs under pathological state. Utoh et al. proposed that high temperature treatment would lead to haemolysis and elevated osmotic fragility of RBCs, which were thought to derive from the changes in composition of membrane^18^. Besides, heat treatment could also result in energy depletion^19^, it was related to the activation of Janus kinase 3 (JAK3) which contributes to the plasma membrane scrambling^20^. Recent research revealed that the effect of temperature was related to accelerated RBC damage/aging^21^. Our research showed that intracellular ROS, Ca^2+^ concentration and PS externalization were increased in stressed RBCs (Fig. 1C–H), these changes were also observed in RBCs of patients with various chronic diseases, such as diabetes^22^, autoimmune haemolytic anaemia^23^, haemolytic uremic syndrome^5^. Nuclei of RBCs are extruded before they enter blood circulation, and there are no organelles such as endoplasmic reticulum and mitochondria in mature RBCs. Thus, RBCs are highly sensitive to changes of internal environment. In some diseases^5,22–24^, hyperosmolar and oxidative stress as well as energy depletion induce changes in RBC membrane surface molecules and intracellular components^2^. Oxidative stress leads to an imbalance of lipid oxidation, intracellular peroxide accumulation, and elevated ROS level^25^. Concurrently, ion channels are aberrantly activated and Ca^2+^ influx increased, resulting in increased intracellular Ca^2+^ concentration, which is related to the translocation of PS from inner leaflet of the cell membrane to the RBC surface^26^. Thus, we chose ROS, Ca^2+^ concentration, and PS exposure to represent the characteristics of stressed RBCs. In fact, the changes in stressed RBCs also resemble changes observed in senescent^6^ and long-term stored RBCs^27–29^.

RBCs under pathological state tend to be phagocytosed by macrophages^6^. Erythrophagocytosis mainly depends on three pathways. Enhanced PS and band-3 clustering on erythrocytes are the predominant pro-phagocytic signals^30,31^. CD47 acts instead as a “do-not-eat-me” signal, mediated via interacting with SIRPα^32^. In the present study, stressed RBCs showed increased PS externalization, which indicated that erythrophagocytosis of stressed RBCs could be mediated by the PS pathway. The in vitro experiment also demonstrated that stressed RBCs were more likely to be phagocytosed by macrophage (Figs. 2, 3C).

This study used high temperature-induced RBCs (stressed RBCs) to investigate the clearance of red blood cells under pathological conditions, but this does not fully represent the RBCs clearance in vivo. In subsequent studies, we will continue to study the in vivo removal of RBCs, such as long-term stored RBCs, and investigate transfusion efficiency of RBCs since the transfusion efficiency of long-term stored RBCs remains poorly understood.

We used ^18^F-FDG labelled RBCs to measure erythrophagocytosis and organ distribution in vivo, confirmed that stressed RBCs tend to be engulfed and spleen is the main organ for stressed RBCs aggregation. Since stressed RBCs simulate damaged RBCs in pathologic conditions, stressed RBCs trapped in spleen may explain the anaemia of some diseases like diabetes and autoimmune haemolytic anaemia^22,23^. Besides, this research laid a foundation for future study on stored RBCs transfusion efficiency in clinical.

## Materials and methods

### Stressed RBCs preparation

Adult 8-week-old BALB/c mice were purchased from the Laboratory Animal Department of Central South University. All animal experiments were approved by the Institutional Animal Care and Use Committees of Xiangya Hospital and Central South University. All the animal experiments were carried out incompliance with the ARRIVE guidelines. All methods were carried out in the accordance with relevant guidelines and regulations. Isolating RBCs from the blood of BALB/c mice was performed as described previously^9^. RBCs were incubated for 30 min at 48 °C to induce stressed RBCs, while untreated RBCs were not.

### Measurement of reactive oxygen species (ROS), intracellular calcium (Ca^2+^) concentration, phosphatidylserine (PS) externalization

To measure ROS, RBCs were mixed with DCFH-DA (Beyotime, China, S0033). To measure intracellular Ca^2+^ concentration, RBCs were mixed with Flou-4 AM (Beyotime, China, S1060). For measurement of PS exposure, RBCs were incubated with FITC-Annexin-V (BD Biosciences, USA, 556547). All reactions were protected from light. RBCs volume was estimated from forward scatter signal. FACS was performed by flow cytometry (BD Biosciences, USA, FACSCantoII). Data were analysed by FlowJo X (Tree Star Inc., USA).

### Detection of erythrophagocytosis

#### Measurement of erythrophagocytosis of RBCs labelled with fluorochrome

RBCs were mixed with TER-119 probe (BD Biosciences, USA, 557915) and incubated on ice for 30 min. Raw 264.7 cells were incubated with TER-119-labelled packed RBCs for 2 h. RBC lysis buffer (Beyotime, China, C3702) was added to remove unphagocytic RBCs. The erythrophagocytosis rate was assessed by FACS. Erythrophagocytosis was observed directly with a fluorescence microscope (Carl Zeiss, Germany, AxioScope.A1) and the images were analysed by ImageJ.

#### Measurement of erythrophagocytosis of RBCs labelled with ^18^F-FDG

RBCs were incubated at 37 °C for 1 h to reduce the intracellular glucose concentration. ^18^F-FDG was provided by Department of PET Center of Xiangya Hospital of Central South University. RBCs were mixed with osmotic pressure-adjusted ^18^F-FDG solution and incubated at 37 °C for 30 min. Then, RBCs were washed with PBS to eliminate extracellular ^18^F-FDG. The radioactivity of the whole suspension and RBCs was measured with a dose calibrator. The labelling efficiency was calculated as a percentage by dividing the post-wash radioactivity of RBCs by the whole-suspension radioactivity. The post-labelling stability was calculated as the RBCs radioactivity by the radioactivity of whole-suspension radioactivity^33^.

^18^F-FDG-labelled RBCs were incubated with Raw 264.7 cells, cultured for 2 h. The supernatant was collected. After the addition of RBC Lysis Buffer, the lysate and Raw 264.7 cells were collected. Erythrophagocytosis was represented as a percentage by dividing the radioactivity ratio of Raw 264.7 cells by the sum radioactivity of Raw 264.7 cell, lysate, and supernatant^34,35^.

### PET/CT imaging and analysis

^18^F-FDG-labelled RBCs were injected via the lateral tail vein. Mice were anesthetized and maintained in anaesthesia state by isoflurane throughout imaging procedure. Imaging was performed using nanoScan PET/CT system (Mediso, Hungary). Image acquisition was started after intravenous injection and lasted for 2 h.

The reconstructed images were analysed by Nucline NanoScan 3.00.018.0000. In the images of BALB/c mice, volume of interest (VOI) was placed in each organ, and the radioactivity was expressed as the percentage of injected dose per gram (%ID/g)^36^. Time-activity curves of cardiac, liver, spleen, lung, and kidney were obtained.

### Haematoxylin and Eosin (H&E) staining and Immunofluorescence

Spleen were separated at 2 h post-transfusion as described previously^9^. Spleens were fixed with 4% paraformaldehyde overnight and embedded in paraffin before sectioning. Sections were stained with haematoxylin and eosin or were deparaffinized and immunostained with anti-mouse TER-119 antibody(BD Biosciences, USA, 550565).

### Statistical analysis

Statistical analysis was performed using SPSS20.0 (SPSS, Inc., Chicago, IL, USA). Data were showed as mean ± standard deviation (SD). Statistical differences were measured by independent sample Student’s t-test and nonparametric test. Differences with *P* values less than 0.05 were considered statistically significant.

### Ethical standards

All methods were carried out in the accordance with relevant guidelines and regulations. All animal experiments were approved by the Institutional Animal Care and Use Committees of Xiangya Hospital and Central South University. All the animal experiments were carried out incompliance with the ARRIVE guidelines.
