# Author Correction: ^18^F-fluorodeoxyglucose positron emission tomography–computed tomography for assessing organ distribution of stressed red blood cells in mice

**DOI:** 10.1038/s41598-021-97280-w

**Published:** 2021-09-06

**Authors:** Wen-yu Yin, Jiao Yuan, Zhi-min Zhang, Cheng Mei, Wei Xu, Yong-xiang Tang, Fang Peng, Ning Li

**Affiliations:** 1 Department of Blood Transfusion, Clinical Transfusion Research Center, Xiangya Hospital, Central South University, Changsha, 410008 Hunan Province China; 2 Department of Infectious Diseases, Xiangya Hospital, Central South University, Changsha, 410008 Hunan Province China; 3 Clinical Laboratory, Xiangya Hospital, Central South University, Changsha, 410008 Hunan Province China; 4 Department of PET Center, Xiangya Hospital, Central South University, Changsha, 410008 Hunan Province China; 5 NHC Key Laboratory of Cancer Proteomics, Xiangya Hospital, Central South University, Changsha, 410008 Hunan Province China

Correction to: *Scientific Reports* 10.1038/s41598-021-82100-y, published online 28 January 2021

The original version of this Article contained an error in Figure 4, where panels 4A and 4B were interchanged.

The original Figure 4 and accompanying legend appear below. The original Article has been corrected.

**Figure 4:**
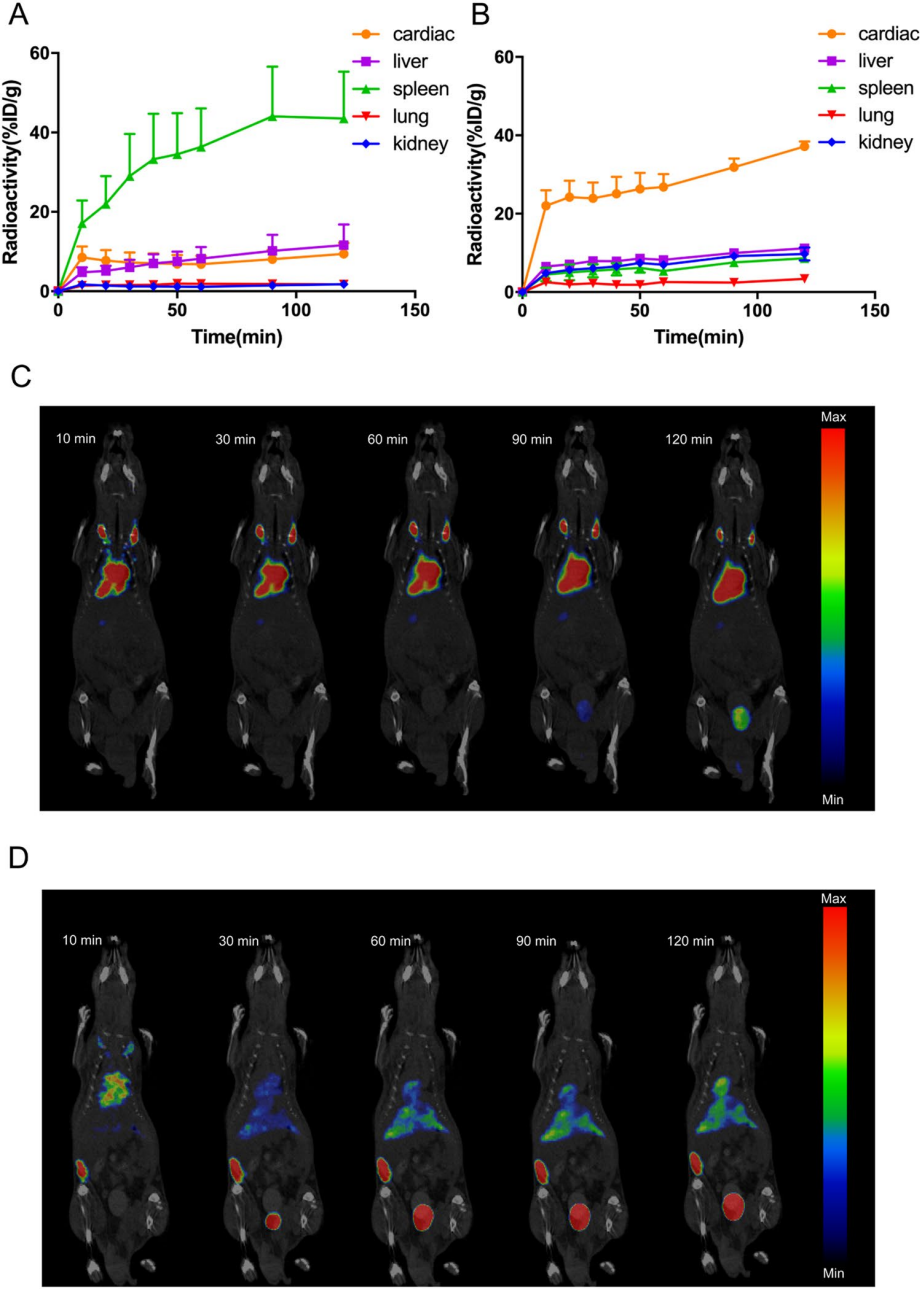
Stressed RBCs were trapped in spleen. (**A**,**B**) Time-activity curves of cardiac, liver, spleen, lung, and kidney of mice injected with ^18^F-FDG-labelled untreated RBCs (**A**) and stressed RBCs (**B**) during PET/CT imaging for 120 min (n = 3); (**C**,**D**) Representative serial images after intravenous injection for 120 min of ^18^F-FDG-labelled untreated RBCs (**C**) and stressed RBCs (**D**).

